# Antimicrobial Resistance in Bacteria of Animal Origin

**DOI:** 10.3201/eid1207.060503

**Published:** 2006-07

**Authors:** Stacy Holzbauer, Tom Chiller

**Affiliations:** * Centers for Disease Control and Prevention, Atlanta, Georgia, USA

**Keywords:** antimicrobial resistance, bacteria, Frank M. Aarestrup

Resistance to antimicrobial agents develops soon after these life-saving drugs are introduced into human and animal medicine. The role of veterinary and animal use of antimicrobial agents has been debated for years. Frank Aarestrup and colleagues attempt to summarize information concerning this topic in their new book, Antimicrobial Resistance in Bacteria of Animal Origin. This book has 51 contributors, who have written 25 chapters on the public health, clinical, and regulatory importance of antimicrobial drug resistance in bacteria of animal origin. The editor recognizes the complexity of this subject and makes no claims to cover all the issues but rather highlights what he and the contributors believe to be the most important topics.

The first 6 chapters highlight modes of action and resistance, history of usage, susceptibility testing, antimicrobial-drug resistance for antimicrobial agents, detection methods, dosing schedules, and mechanisms that lead to the spread of bacterial resistance. These chapters provide the reader with very detailed molecular and genetic information on resistance mechanisms in bacteria of animal origin. Knowing the pharmacodynamics and pharmacokinetics of antimicrobial agents is essential for these drugs to be used correctly, and a good overview of these mechanisms is also provided in these beginning chapters. The book also stresses the urgent need for establishing veterinary-validated breakpoints for species-specific host-pathogen combinations that are clinically relevant. Some of the tables and diagrams in these chapters contain a large amount of material and need to be read carefully to understand the total wealth of information.

The 12 middle chapters provide an in-depth review of the known resistance mechanisms found in most of the pathogenic bacteria and bacteria of public health importance in animals. Each chapter takes a closer look at a particular family, genus, or species of bacteria and, when possible, attempts to estimate the prevalence of resistance to key antimicrobial agents. The information provided in these chapters is useful to clinicians, researchers, public health officials, and regulators. For some zoonotic agents, the animal health consequences of resistance are not known. For future editions, expanding on this topic would be helpful.

The last 7 chapters attempt to tie all of the previous information together by providing an overview of the licensing and approval procedures for veterinary antimicrobial agents, surveillance systems that monitor resistance and usage, and the use of risk assessments to guide industry and government in decision making. These chapters take a global approach. When possible, side-by-side comparisons of resistance data or surveillance systems are discussed.

This book is the first of its kind to provide a comprehensive overview of resistance mechanism in bacteria of animal origin rather than concentrating solely on zoonotic or foodborne bacteria. All uses of antimicrobial agents contribute to resistance, and each use must be examined in an attempt to understand its part in encouraging further dissemination of resistance in bacteria, including bacteria of animal origin. This book will serve as a valuable reference for persons who treat, research, or monitor resistance in bacteria of animal origin.

